# Methyl 3,5-bis­[(4-hydroxy­methyl-2-methoxy­phen­oxy)meth­yl]benzoate

**DOI:** 10.1107/S160053681000677X

**Published:** 2010-02-27

**Authors:** Muhammad Nadeem Arshad, Scott T. Mough, John C. Goeltz, K. Travis Holman

**Affiliations:** a Department of Chemistry, GC University Lahore 54000, Pakistan; b Department of Chemistry, Georgetown University, 37th and O St NW, Washington, DC 20057, USA

## Abstract

In the title compound, C_26_H_28_O_8_, the central aromatic ring forms dihedral angles of 24.32 (11) and 80.19 (7)° with the two adjoining vanillyl alcohol rings. In the crystal, O—H⋯O hydrogen bonds connect the mol­ecules, forming a hydrogen-bonded sheet-like motif extended in the *ab* plane.

## Related literature

For the synthesis of and background to adjoined vanillyl alcohols, see: Mough *et al.* (2004 ▶); Mough & Holman (2008 ▶). For background to cryptophanes, see: Brotin & Dutasta (2009 ▶).
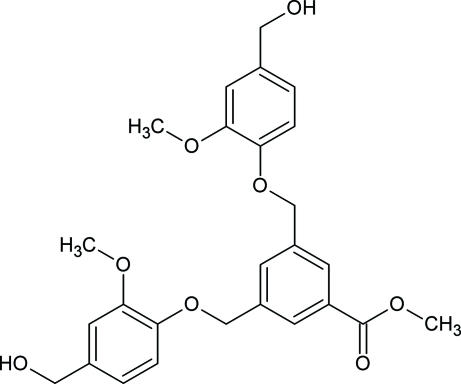


## Experimental

### 

#### Crystal data

C_26_H_28_O_8_
*M* *_r_* = 468.48
Triclinic, 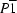

*a* = 4.7707 (12) Å
*b* = 14.844 (4) Å
*c* = 16.349 (4) Å
α = 99.801 (5)°
β = 95.692 (5)°
γ = 92.821 (5)°
*V* = 1132.7 (5) Å^3^
*Z* = 2
Mo *K*α radiation
μ = 0.10 mm^−1^
*T* = 173 K
0.50 × 0.25 × 0.05 mm

#### Data collection

Bruker SMART 1K diffractometer
Absorption correction: multi-scan (*SADABS*; Bruker, 2001 ▶) *T*_min_ = 0.951, *T*_max_ = 0.995
6559 measured reflections
4394 independent reflections
2197 reflections with *I* > 2σ(*I*)
*R*_int_ = 0.037

#### Refinement

*R*[*F* ^2^ > 2σ(*F* ^2^)] = 0.054
*wR*(*F* ^2^) = 0.128
*S* = 0.85
4394 reflections
312 parameters
H-atom parameters constrained
Δρ_max_ = 0.40 e Å^−3^
Δρ_min_ = −0.22 e Å^−3^

### 

Data collection: *SMART* (Bruker, 2001 ▶); cell refinement: *SAINT* (Bruker, 2001 ▶); data reduction: *SAINT*; program(s) used to solve structure: *SHELXS97* (Sheldrick, 2008 ▶); program(s) used to refine structure: *SHELXL97* (Sheldrick, 2008 ▶); molecular graphics: *ORTEP-3* (Farrugia, 1997 ▶), *PLATON* (Spek, 2009 ▶) and *X-SEED* (Barbour, 2001 ▶); software used to prepare material for publication: *WinGX* (Farrugia, 1999 ▶) and *PLATON*.

## Supplementary Material

Crystal structure: contains datablocks global, I. DOI: 10.1107/S160053681000677X/ng2730sup1.cif

Structure factors: contains datablocks I. DOI: 10.1107/S160053681000677X/ng2730Isup2.hkl

Additional supplementary materials:  crystallographic information; 3D view; checkCIF report

## Figures and Tables

**Table 1:** Hydrogen-bond geometry (Å, °)

*D*—H⋯*A*	*D*—H	H⋯*A*	*D*⋯*A*	*D*—H⋯*A*
O3—H3⋯O8^i^	0.84	1.90	2.731 (3)	170
O8—H8⋯O3^ii^	0.84	1.90	2.721 (3)	167

Symmetry codes: (i) 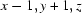; (ii) 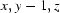.

**Fig. 1.**
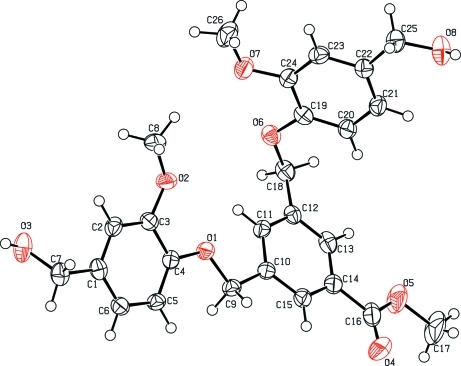
A thermal ellisoid plot of I, at 50% probability.

**Fig. 2.**
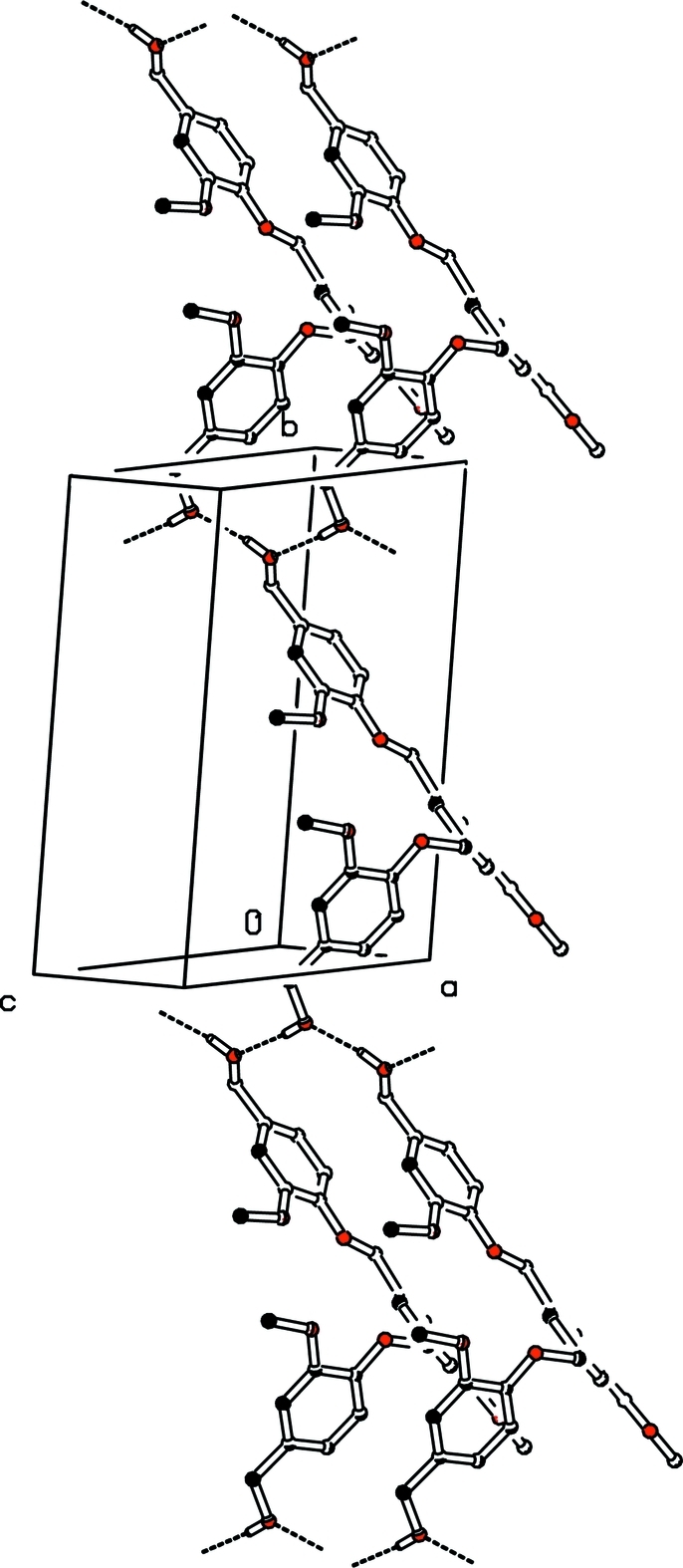
Unit cell packing diagram of I depicting the hydrogen bonds as dashed lines. Hydrogen atoms not involved in O—H···O hydrogen bonding have been omitted for clarity.

## supplementary crystallographic information

### Comment

Adjoined vanillyl alcohols have been used extensively in the synthesis of container-like host molecules known as cryptophanes (Brotin & Dutasta, 2009). Our group has used the title compound as a precursor for the synthesis of a *m*-xylyl bridged cryptophane (Mough *et al.*, 2004) that displays uncommon conformational behavior and whose carboxylic acid derivative has been used as a ligand for the synthesis of coordination polymers possessing container-like components (Mough *et al.*, 2008).

The title compounds consists of two vanillyl alcohol moieties linked by a 3,5 disubstituted methylbenzoate. The arene rings of the vanilliyl alcohol moieties A (C1/C2/C3/C4/C5/C6) and B (C19/C20/C21/C22/C23/C24) are oriented, respectively, at dihedral angles of 24.32(0.11)° and 80.19(0.07)° with respect to the central methyl benzoate ring C (C10/C11/C12/C13/C14/C15). The hydroxyl groups available at each end of molecule participate in chains of O–H···O type hydrogen bonds that extend along the *a*-axis in the crystal (Table 1, Fig. 2). The molecules are thus connected into a hydrogen bonded polymeric sheet that resides in the *ab* plane.

### Experimental

The compound was prepared following the method of Mough *et al.* (2004).

### Refinement

All the C–H and O–H, H-atoms were positioned geometrically and refined using a riding model with: d(C—H) = 0.95Å and 0.99 Å, U_iso_ = 1.2U_eq_ (C) for aromatic and methylene C atoms, d(C—H)=0.98Å U_iso_ = 1.5U_eq_ (C) for methyl, d(C—H)=0.84Å U_iso_ = 1.2U_eq_ (O) for Hydroxyl.

### Crystal data

**Table:**

C_26_H_28_O_8_	*Z* = 2
*M**_r_* = 468.48	*F*(000) = 496
Triclinic, *P*1	*D*_x_ = 1.374 Mg m^−^^3^
Hall symbol: -P 1	Mo *K*α radiation, λ = 0.71073 Å
*a* = 4.7707 (12) Å	Cell parameters from 1285 reflections
*b* = 14.844 (4) Å	θ = 2.5–24.5°
*c* = 16.349 (4) Å	µ = 0.10 mm^−^^1^
α = 99.801 (5)°	*T* = 173 K
β = 95.692 (5)°	Needle, pale yellow
γ = 92.821 (5)°	0.50 × 0.25 × 0.05 mm
*V* = 1132.7 (5) Å^3^	

### Data collection

**Table:**

Bruker SMART K1 diffractometer	4394 independent reflections
Radiation source: fine-focus sealed tube	2197 reflections with *I* > 2σ(*I*)
graphite	*R*_int_ = 0.037
ω scan	θ_max_ = 26.0°, θ_min_ = 1.7°
Absorption correction: multi-scan (*SADABS*; Bruker, 2001)	*h* = −5→5
*T*_min_ = 0.951, *T*_max_ = 0.995	*k* = −16→18
6559 measured reflections	*l* = −18→20

### Refinement

**Table:**

Refinement on *F*^2^	Primary atom site location: structure-invariant direct methods
Least-squares matrix: full	Secondary atom site location: difference Fourier map
*R*[*F*^2^ > 2σ(*F*^2^)] = 0.054	Hydrogen site location: inferred from neighbouring sites
*wR*(*F*^2^) = 0.128	H-atom parameters constrained
*S* = 0.85	*w* = 1/[σ^2^(*F*_o_^2^) + (0.0568*P*)^2^] where *P* = (*F*_o_^2^ + 2*F*_c_^2^)/3
4394 reflections	(Δ/σ)_max_ < 0.001
312 parameters	Δρ_max_ = 0.40 e Å^−^^3^
0 restraints	Δρ_min_ = −0.22 e Å^−^^3^

### Special details

**Table:**

Geometry. All s.u.'s (except the s.u. in the dihedral angle between two l.s. planes) are estimated using the full covariance matrix. The cell s.u.'s are taken into account individually in the estimation of s.u.'s in distances, angles and torsion angles; correlations between s.u.'s in cell parameters are only used when they are defined by crystal symmetry. An approximate (isotropic) treatment of cell s.u.'s is used for estimating s.u.'s involving l.s. planes.
Refinement. Refinement of *F*^2^ against ALL reflections. The weighted *R*-factor wR and goodness of fit *S* are based on *F*^2^, conventional *R*-factors *R* are based on *F*, with *F* set to zero for negative *F*^2^. The threshold expression of *F*^2^ > 2σ(*F*^2^) is used only for calculating *R*-factors(gt) etc. and is not relevant to the choice of reflections for refinement. *R*-factors based on *F*^2^ are statistically about twice as large as those based on *F*, and *R*- factors based on ALL data will be even larger.

### Fractional atomic coordinates and isotropic or equivalent isotropic displacement parameters (Å^2^)

**Table:**

	*x*	*y*	*z*	*U*_iso_*/*U*_eq_	
O1	0.8577 (4)	0.44795 (11)	0.18004 (10)	0.0323 (5)	
C1	0.3350 (6)	0.66420 (18)	0.21353 (17)	0.0325 (7)	
O2	0.6676 (4)	0.49101 (12)	0.32243 (10)	0.0400 (5)	
C2	0.4117 (6)	0.61493 (18)	0.27658 (17)	0.0329 (7)	
H2	0.3434	0.6310	0.3294	0.039*	
O3	0.2404 (4)	0.80315 (13)	0.30289 (13)	0.0490 (6)	
H3	0.1081	0.8346	0.3191	0.059*	
C3	0.5843 (6)	0.54346 (18)	0.26403 (16)	0.0319 (7)	
O4	1.3168 (6)	0.15167 (15)	−0.10157 (14)	0.0725 (8)	
C4	0.6859 (5)	0.51921 (17)	0.18579 (16)	0.0285 (6)	
O5	1.6491 (5)	0.09734 (14)	−0.02106 (13)	0.0614 (7)	
C5	0.6078 (5)	0.56715 (18)	0.12281 (16)	0.0299 (7)	
H5	0.6727	0.5508	0.0695	0.036*	
O6	1.4405 (4)	0.26847 (12)	0.34342 (10)	0.0352 (5)	
C6	0.4340 (6)	0.63941 (17)	0.13746 (16)	0.0319 (7)	
H6	0.3828	0.6723	0.0939	0.038*	
O7	1.1515 (4)	0.28885 (12)	0.46990 (11)	0.0436 (5)	
C7	0.1416 (6)	0.74113 (18)	0.22818 (17)	0.0409 (8)	
H7A	0.1326	0.7742	0.1803	0.049*	
H7B	−0.0512	0.7157	0.2324	0.049*	
O8	0.7672 (4)	−0.11335 (12)	0.34750 (13)	0.0489 (6)	
H8	0.6119	−0.1407	0.3263	0.059*	
C8	0.5065 (7)	0.4943 (2)	0.39253 (16)	0.0499 (9)	
H8A	0.5503	0.5532	0.4303	0.075*	
H8B	0.5547	0.4443	0.4224	0.075*	
H8C	0.3045	0.4877	0.3727	0.075*	
C9	0.9429 (6)	0.41381 (17)	0.10055 (15)	0.0304 (7)	
H9A	1.0410	0.4637	0.0789	0.036*	
H9B	0.7755	0.3897	0.0607	0.036*	
C10	1.1380 (6)	0.33864 (17)	0.10909 (16)	0.0286 (6)	
C11	1.2673 (5)	0.32889 (17)	0.18610 (16)	0.0283 (6)	
H11	1.2218	0.3673	0.2350	0.034*	
C12	1.4638 (5)	0.26345 (17)	0.19325 (16)	0.0287 (6)	
C13	1.5267 (6)	0.20702 (18)	0.12191 (17)	0.0351 (7)	
H13	1.6623	0.1628	0.1261	0.042*	
C14	1.3922 (6)	0.21470 (18)	0.04403 (17)	0.0373 (7)	
C15	1.1980 (6)	0.28043 (17)	0.03803 (17)	0.0327 (7)	
H15	1.1055	0.2856	−0.0150	0.039*	
C16	1.4431 (8)	0.1529 (2)	−0.0346 (2)	0.0472 (9)	
C17	1.7031 (9)	0.0358 (2)	−0.0969 (2)	0.0867 (14)	
H17A	1.5272	0.0010	−0.1226	0.130*	
H17B	1.8436	−0.0066	−0.0827	0.130*	
H17C	1.7746	0.0719	−0.1363	0.130*	
C18	1.6184 (6)	0.25862 (18)	0.27714 (16)	0.0357 (7)	
H18A	1.7048	0.1990	0.2739	0.043*	
H18B	1.7730	0.3075	0.2904	0.043*	
C19	1.2662 (5)	0.19350 (18)	0.34944 (16)	0.0300 (7)	
C20	1.2295 (6)	0.11288 (18)	0.29360 (16)	0.0338 (7)	
H20	1.3278	0.1060	0.2454	0.041*	
C21	1.0492 (6)	0.04030 (18)	0.30645 (16)	0.0353 (7)	
H21	1.0240	−0.0149	0.2666	0.042*	
C22	0.9088 (6)	0.04871 (18)	0.37649 (16)	0.0310 (7)	
C23	0.9420 (6)	0.13196 (18)	0.43279 (16)	0.0346 (7)	
H23	0.8427	0.1388	0.4808	0.042*	
C24	1.1169 (6)	0.20460 (18)	0.41988 (16)	0.0318 (7)	
C25	0.7190 (6)	−0.02789 (18)	0.39519 (17)	0.0374 (7)	
H25A	0.7527	−0.0311	0.4553	0.045*	
H25B	0.5194	−0.0145	0.3830	0.045*	
C26	0.9726 (7)	0.3043 (2)	0.53515 (18)	0.0574 (10)	
H26A	0.7749	0.2921	0.5113	0.086*	
H26B	1.0032	0.3681	0.5639	0.086*	
H26C	1.0167	0.2634	0.5750	0.086*	

### Atomic displacement parameters (Å^2^)

**Table:**

	*U*^11^	*U*^22^	*U*^33^	*U*^12^	*U*^13^	*U*^23^
O1	0.0385 (12)	0.0315 (11)	0.0290 (10)	0.0110 (9)	0.0096 (9)	0.0052 (8)
C1	0.0261 (16)	0.0276 (16)	0.0426 (17)	−0.0002 (12)	0.0008 (14)	0.0049 (13)
O2	0.0550 (14)	0.0404 (12)	0.0295 (10)	0.0163 (10)	0.0141 (10)	0.0110 (9)
C2	0.0346 (17)	0.0310 (17)	0.0326 (16)	0.0031 (13)	0.0112 (14)	−0.0002 (13)
O3	0.0348 (13)	0.0368 (13)	0.0694 (14)	0.0063 (10)	0.0067 (11)	−0.0090 (11)
C3	0.0349 (17)	0.0292 (17)	0.0333 (16)	0.0034 (13)	0.0063 (14)	0.0086 (13)
O4	0.120 (2)	0.0577 (16)	0.0399 (14)	0.0244 (15)	0.0195 (15)	−0.0019 (12)
C4	0.0276 (16)	0.0265 (16)	0.0308 (15)	−0.0001 (12)	0.0068 (13)	0.0020 (12)
O5	0.0712 (17)	0.0463 (14)	0.0649 (15)	0.0127 (12)	0.0290 (14)	−0.0101 (12)
C5	0.0316 (17)	0.0332 (16)	0.0256 (14)	0.0006 (13)	0.0071 (13)	0.0050 (12)
O6	0.0340 (12)	0.0325 (12)	0.0412 (11)	0.0042 (9)	0.0086 (10)	0.0091 (9)
C6	0.0339 (17)	0.0299 (16)	0.0324 (16)	0.0017 (13)	0.0022 (14)	0.0080 (13)
O7	0.0512 (14)	0.0371 (12)	0.0403 (11)	−0.0025 (10)	0.0139 (11)	−0.0028 (10)
C7	0.0418 (19)	0.0323 (17)	0.0476 (18)	0.0070 (14)	0.0039 (16)	0.0034 (14)
O8	0.0357 (13)	0.0330 (12)	0.0749 (15)	0.0017 (9)	0.0107 (12)	−0.0023 (11)
C8	0.079 (3)	0.044 (2)	0.0330 (16)	0.0115 (17)	0.0238 (17)	0.0128 (14)
C9	0.0321 (16)	0.0332 (16)	0.0266 (14)	0.0019 (13)	0.0064 (13)	0.0058 (12)
C10	0.0297 (16)	0.0279 (16)	0.0292 (15)	−0.0016 (12)	0.0101 (13)	0.0048 (12)
C11	0.0279 (16)	0.0285 (16)	0.0285 (15)	−0.0008 (12)	0.0090 (13)	0.0020 (12)
C12	0.0264 (16)	0.0254 (15)	0.0359 (16)	0.0016 (12)	0.0084 (13)	0.0072 (13)
C13	0.0308 (17)	0.0282 (16)	0.0499 (18)	0.0056 (13)	0.0171 (15)	0.0083 (14)
C14	0.047 (2)	0.0292 (17)	0.0381 (17)	−0.0022 (14)	0.0216 (16)	0.0051 (14)
C15	0.0369 (18)	0.0280 (16)	0.0338 (16)	−0.0013 (13)	0.0102 (14)	0.0045 (13)
C16	0.061 (2)	0.0284 (18)	0.055 (2)	0.0008 (16)	0.027 (2)	0.0027 (17)
C17	0.115 (4)	0.055 (2)	0.088 (3)	0.012 (2)	0.059 (3)	−0.022 (2)
C18	0.0301 (17)	0.0334 (17)	0.0472 (18)	0.0090 (13)	0.0123 (15)	0.0106 (14)
C19	0.0228 (16)	0.0320 (17)	0.0370 (16)	0.0043 (13)	0.0029 (13)	0.0111 (13)
C20	0.0371 (18)	0.0314 (17)	0.0349 (15)	0.0086 (14)	0.0095 (14)	0.0065 (13)
C21	0.0398 (18)	0.0298 (17)	0.0351 (16)	0.0089 (14)	0.0039 (14)	0.0010 (13)
C22	0.0271 (16)	0.0317 (17)	0.0341 (15)	0.0049 (13)	0.0011 (13)	0.0062 (13)
C23	0.0349 (17)	0.0405 (18)	0.0302 (15)	0.0078 (14)	0.0069 (13)	0.0080 (13)
C24	0.0312 (17)	0.0320 (17)	0.0310 (15)	0.0037 (13)	0.0016 (13)	0.0025 (13)
C25	0.0323 (17)	0.0338 (18)	0.0459 (17)	0.0051 (13)	0.0080 (15)	0.0032 (14)
C26	0.071 (3)	0.050 (2)	0.0486 (19)	−0.0013 (18)	0.0276 (19)	−0.0092 (16)

### Geometric parameters (Å, °)

**Table:**

O1—C4	1.366 (3)	C9—H9B	0.9900
O1—C9	1.418 (3)	C10—C11	1.380 (3)
C1—C6	1.372 (3)	C10—C15	1.388 (3)
C1—C2	1.393 (4)	C11—C12	1.394 (4)
C1—C7	1.506 (4)	C11—H11	0.9500
O2—C3	1.372 (3)	C12—C13	1.383 (3)
O2—C8	1.437 (3)	C12—C18	1.506 (4)
C2—C3	1.375 (4)	C13—C14	1.393 (4)
C2—H2	0.9500	C13—H13	0.9500
O3—C7	1.422 (3)	C14—C15	1.387 (4)
O3—H3	0.8400	C14—C16	1.495 (4)
C3—C4	1.409 (3)	C15—H15	0.9500
O4—C16	1.193 (4)	C17—H17A	0.9800
C4—C5	1.380 (3)	C17—H17B	0.9800
O5—C16	1.339 (4)	C17—H17C	0.9800
O5—C17	1.462 (3)	C18—H18A	0.9900
C5—C6	1.391 (4)	C18—H18B	0.9900
C5—H5	0.9500	C19—C20	1.368 (3)
O6—C19	1.379 (3)	C19—C24	1.404 (3)
O6—C18	1.434 (3)	C20—C21	1.402 (3)
C6—H6	0.9500	C20—H20	0.9500
O7—C24	1.366 (3)	C21—C22	1.374 (3)
O7—C26	1.427 (3)	C21—H21	0.9500
C7—H7A	0.9900	C22—C23	1.401 (3)
C7—H7B	0.9900	C22—C25	1.509 (3)
O8—C25	1.413 (3)	C23—C24	1.388 (4)
O8—H8	0.8400	C23—H23	0.9500
C8—H8A	0.9800	C25—H25A	0.9900
C8—H8B	0.9800	C25—H25B	0.9900
C8—H8C	0.9800	C26—H26A	0.9800
C9—C10	1.503 (4)	C26—H26B	0.9800
C9—H9A	0.9900	C26—H26C	0.9800
			
C4—O1—C9	117.7 (2)	C12—C13—H13	119.8
C6—C1—C2	118.4 (2)	C14—C13—H13	119.8
C6—C1—C7	121.0 (3)	C15—C14—C13	119.7 (3)
C2—C1—C7	120.5 (2)	C15—C14—C16	117.7 (3)
C3—O2—C8	116.9 (2)	C13—C14—C16	122.6 (3)
C3—C2—C1	121.4 (2)	C14—C15—C10	120.3 (3)
C3—C2—H2	119.3	C14—C15—H15	119.8
C1—C2—H2	119.3	C10—C15—H15	119.8
C7—O3—H3	109.5	O4—C16—O5	123.3 (3)
O2—C3—C2	125.1 (2)	O4—C16—C14	125.1 (3)
O2—C3—C4	115.4 (2)	O5—C16—C14	111.6 (3)
C2—C3—C4	119.5 (3)	O5—C17—H17A	109.5
O1—C4—C5	125.8 (2)	O5—C17—H17B	109.5
O1—C4—C3	114.9 (2)	H17A—C17—H17B	109.5
C5—C4—C3	119.3 (2)	O5—C17—H17C	109.5
C16—O5—C17	112.9 (3)	H17A—C17—H17C	109.5
C4—C5—C6	119.9 (2)	H17B—C17—H17C	109.5
C4—C5—H5	120.0	O6—C18—C12	113.4 (2)
C6—C5—H5	120.0	O6—C18—H18A	108.9
C19—O6—C18	117.38 (19)	C12—C18—H18A	108.9
C1—C6—C5	121.4 (3)	O6—C18—H18B	108.9
C1—C6—H6	119.3	C12—C18—H18B	108.9
C5—C6—H6	119.3	H18A—C18—H18B	107.7
C24—O7—C26	116.3 (2)	C20—C19—O6	125.7 (2)
O3—C7—C1	110.5 (2)	C20—C19—C24	119.6 (2)
O3—C7—H7A	109.5	O6—C19—C24	114.6 (2)
C1—C7—H7A	109.5	C19—C20—C21	121.0 (2)
O3—C7—H7B	109.5	C19—C20—H20	119.5
C1—C7—H7B	109.5	C21—C20—H20	119.5
H7A—C7—H7B	108.1	C22—C21—C20	120.2 (2)
C25—O8—H8	109.5	C22—C21—H21	119.9
O2—C8—H8A	109.5	C20—C21—H21	119.9
O2—C8—H8B	109.5	C21—C22—C23	118.7 (3)
H8A—C8—H8B	109.5	C21—C22—C25	122.7 (2)
O2—C8—H8C	109.5	C23—C22—C25	118.6 (2)
H8A—C8—H8C	109.5	C24—C23—C22	121.4 (2)
H8B—C8—H8C	109.5	C24—C23—H23	119.3
O1—C9—C10	108.8 (2)	C22—C23—H23	119.3
O1—C9—H9A	109.9	O7—C24—C23	124.9 (2)
C10—C9—H9A	109.9	O7—C24—C19	116.1 (2)
O1—C9—H9B	109.9	C23—C24—C19	119.0 (2)
C10—C9—H9B	109.9	O8—C25—C22	111.7 (2)
H9A—C9—H9B	108.3	O8—C25—H25A	109.3
C11—C10—C15	119.4 (3)	C22—C25—H25A	109.3
C11—C10—C9	121.0 (2)	O8—C25—H25B	109.3
C15—C10—C9	119.5 (2)	C22—C25—H25B	109.3
C10—C11—C12	121.0 (2)	H25A—C25—H25B	107.9
C10—C11—H11	119.5	O7—C26—H26A	109.5
C12—C11—H11	119.5	O7—C26—H26B	109.5
C13—C12—C11	119.2 (2)	H26A—C26—H26B	109.5
C13—C12—C18	120.6 (2)	O7—C26—H26C	109.5
C11—C12—C18	120.1 (2)	H26A—C26—H26C	109.5
C12—C13—C14	120.4 (3)	H26B—C26—H26C	109.5
			
C6—C1—C2—C3	0.5 (4)	C16—C14—C15—C10	178.8 (2)
C7—C1—C2—C3	178.9 (3)	C11—C10—C15—C14	−1.8 (4)
C8—O2—C3—C2	17.4 (4)	C9—C10—C15—C14	175.6 (2)
C8—O2—C3—C4	−161.8 (2)	C17—O5—C16—O4	0.3 (4)
C1—C2—C3—O2	−179.3 (2)	C17—O5—C16—C14	179.8 (2)
C1—C2—C3—C4	−0.1 (4)	C15—C14—C16—O4	−5.0 (4)
C9—O1—C4—C5	−6.5 (4)	C13—C14—C16—O4	173.5 (3)
C9—O1—C4—C3	173.5 (2)	C15—C14—C16—O5	175.5 (2)
O2—C3—C4—O1	−1.3 (3)	C13—C14—C16—O5	−6.0 (4)
C2—C3—C4—O1	179.4 (2)	C19—O6—C18—C12	77.3 (3)
O2—C3—C4—C5	178.7 (2)	C13—C12—C18—O6	−141.4 (2)
C2—C3—C4—C5	−0.6 (4)	C11—C12—C18—O6	42.5 (3)
O1—C4—C5—C6	−179.1 (2)	C18—O6—C19—C20	−6.0 (4)
C3—C4—C5—C6	1.0 (4)	C18—O6—C19—C24	174.4 (2)
C2—C1—C6—C5	−0.2 (4)	O6—C19—C20—C21	179.1 (3)
C7—C1—C6—C5	−178.6 (2)	C24—C19—C20—C21	−1.3 (4)
C4—C5—C6—C1	−0.5 (4)	C19—C20—C21—C22	−1.0 (4)
C6—C1—C7—O3	−130.8 (3)	C20—C21—C22—C23	2.2 (4)
C2—C1—C7—O3	50.8 (3)	C20—C21—C22—C25	−178.1 (3)
C4—O1—C9—C10	177.9 (2)	C21—C22—C23—C24	−1.3 (4)
O1—C9—C10—C11	−17.5 (3)	C25—C22—C23—C24	179.0 (3)
O1—C9—C10—C15	165.2 (2)	C26—O7—C24—C23	−6.2 (4)
C15—C10—C11—C12	2.1 (4)	C26—O7—C24—C19	172.0 (3)
C9—C10—C11—C12	−175.3 (2)	C22—C23—C24—O7	177.2 (2)
C10—C11—C12—C13	−0.7 (4)	C22—C23—C24—C19	−0.9 (4)
C10—C11—C12—C18	175.5 (2)	C20—C19—C24—O7	−176.1 (2)
C11—C12—C13—C14	−0.9 (4)	O6—C19—C24—O7	3.5 (3)
C18—C12—C13—C14	−177.1 (2)	C20—C19—C24—C23	2.2 (4)
C12—C13—C14—C15	1.1 (4)	O6—C19—C24—C23	−178.1 (2)
C12—C13—C14—C16	−177.4 (2)	C21—C22—C25—O8	16.9 (4)
C13—C14—C15—C10	0.2 (4)	C23—C22—C25—O8	−163.4 (2)

### Hydrogen-bond geometry (Å, °)

**Table:**

*D*—H···*A*	*D*—H	H···*A*	*D*···*A*	*D*—H···*A*
O3—H3···O8^i^	0.84	1.90	2.731 (3)	170
O8—H8···O3^ii^	0.84	1.90	2.721 (3)	167

Symmetry codes: (i) *x*−1, *y*+1, *z*; (ii) *x*, *y*−1, *z*.
